# Role of Responsible Leadership for Organizational Citizenship Behavior for the Environment in Light of Psychological Ownership and Employee Environmental Commitment: A Moderated Mediation Model

**DOI:** 10.3389/fpsyg.2021.756570

**Published:** 2022-02-08

**Authors:** Ali Abbas, Ye Chengang, Sufan Zhuo, Bilal, Shahid Manzoor, Irfan Ullah, Yasir Hayat Mughal

**Affiliations:** ^1^Business School, University of International Business and Economics, Beijing, China; ^2^School of Accounting, Hubei University of Economics, Wuhan, China; ^3^Hailey College of Commerce, University of the Punjab, Lahore, Pakistan; ^4^School of Management and Economics, Beijing Institute of Technology, Beijing, China; ^5^Department of Health Administration, College of Public Health and Health Informatics, Qassim University, Al Bukayriyah, Saudi Arabia

**Keywords:** responsible leadership, psychological ownership, employee environmental commitment, organizational citizenship behavior for the environment, China

## Abstract

The world is looking toward organizations for social responsibility to contribute to a sustainable environment. Employees’ organizational citizenship behavior for the environment (OCBE) is a voluntary environmental-oriented behavior that is important for organizations’ environmental performance. Based on social learning theory, this study examined the effects of responsible leadership in connection with OCBE by using a sample of 520 employees in the manufacturing and service sectors in China including engine manufacturing, petroleum plants, banking, and insurance sector organizations. Further, the roles of psychological ownership and employee environmental commitment were used as mediators and moderators simultaneously. The direct, mediation, and moderation model results exposed a positive relationship between responsible leadership and OCBE *via* employee psychological ownership and employee environmental commitment. The study also revealed that the indirect effect is stronger when employees hold a higher employee environmental commitment. The theoretical and practical implications for environmental sustainability in respect of organizations as well as future research directions are discussed.

## Introduction

Global climate change and biodiversity reduction have increased in recent years, which has raised positive ecological expectations of the corporate sector (Han et al., 2019a). Common environmental problems like water, air, and soil pollution, smog, biodiversity reduction, global warming, and environmental degradation have caused keen concerns (Liu et al., 2020). Among others, carbon emission is the main cause of climate change (Wang B. et al., 2019; Li et al., 2021). China is a major producer of carbon emissions, as most of its energy needs are fulfilled by the consumption of fossils fuels, including coal, oil, and gas. According to Wang B. et al. (2019) the 60–70% energy needs of manufacturing and services sector organizations, including the electricity generation sector, are met by coal consumption, the highest source of carbon emission. Enterprises are using green business activities, such as green human resource management, green supply chain management, green finance, to pursue long-term development (Han et al., 2019a; Afsar et al., 2020). The role of various stakeholders for sustainable development is crucial at all levels of organizations, including public, non-profit, and commercial entities. Environmental and sustainable managements’ contribution has received attention from management academics and policymakers (Bansal and Song, 2017). Nonetheless, while management scholars have paid attention to strategic and operational corporate environmental protection behavior, they have overlooked the vital role of employees’ behavior toward sustainability and environmental protection (Galpin and Whittington, 2012).

Being critical stakeholders of organizations, organizations’ environmental protection behavior is highly dependent upon behaviors and interpersonal interaction of employees of any enterprise (Felin et al., 2015). It is why the study of the behavior of employees directed toward the environment, known as organizational citizenship behavior for the environment (OCBE), is of significant prominence. Organizational citizenship behavior for the environment is a set of voluntary environmental-oriented activities and practices of employees within the organization that is not covered under any formal reward system (Daily et al., 2009). Explicitly, individual employees engage in environmentally friendly behavior and formulate environmentally friendly concepts consistent with green organizational strategy, such as saving paper, reducing energy consumption, making recommendations, and helping colleagues engage in environmental protective behaviors (Ramus and Steger, 2000).

The substantial impact of employee environmental protective behavior and outcomes has driven attention toward the factors that encourage OCBE. These factors include the self-responsibility of employees for environmental protection (Zhang et al., 2016), and corporate environmental concerns (Temminck et al., 2015), approaches, and attitudes (Lamm et al., 2015). OCBE is an extra-role activity beyond the assigned job duties to improve and protect the organization’s environmental performance (Han et al., 2019a; Ullah et al., 2021). Leadership affects organizational citizenship behavior for the environment in the shades of interaction between leaders and employees within an organization (Han et al., 2019b). This is why we are examining the impact of leaders on employees’ environmental behavior: the role of all the stakeholders cannot be neglected. Responsible leadership, a combination of leadership and social responsibility, is a leadership style that concentrates upon various stakeholders’ interests, including employees, and works to incorporate social, economic, and environmental benefits (Han et al., 2019a; Afsar et al., 2020). It is aligned with the basic idea of OCBE. Several studies have explored the impact of leadership on OCBE (Li et al., 2020), but a few studies have examined the connection between responsible leadership and OCBE (Han et al., 2019a,b; Zhao and Zhou, 2019, 2020; Ullah et al., 2021). Responsible leadership takes environmental concerns as a critical stakeholder that corresponds with OCBE.

This paper significantly contributes to the existing literature on responsible leadership and organizational citizenship behavior for the environment in several ways. First, the rarely investigated relationship of responsible leadership and OCBE is investigated and extended. Responsible leadership undertakes the interest of stakeholders, including employees and their concerns about the environment and their psychological ownership for organizations. In this way, it further enriches the antecedents of OCBE. Second, this study also sheds light on existing models of responsible leadership and OCBE in connection with employees’ psychological ownership and analyzes its mediating effects on the primary relationship of OCBE and responsible leadership. Previously, the leadership role was highlighted regarding OCBE in the mediation of psychological ownership (Jiang et al., 2019; Mi et al., 2019), but the role of responsible leadership was not examined. Thirdly, Tuan (2019) and Pham et al. (2020) examined employee environmental commitment with environmentally specific charismatic leadership but did not explore the relationship between responsible leadership and OCBE. To fill this gap, responsible leadership and psychological ownership are further tested with the moderating role of employee environmental commitments, and thus this study adds to the existing body of knowledge. Fourth, much of the previous OCBE research has been carried out in the western context. However, this study was carried out in China, which has many environmental concerns. The rest of the article is distributed as follows: literature review and hypothesis development in the next section followed by methodology of the article. The results and findings of the study are discussed in the next part, further discussion, and the practical implications, limitations and suggestions are discussed in the last.

## Theory and Hypothesis Development

### Responsible Leadership

Responsible leadership originated from social relations and ethical theories and a leadership style that took place in the social interaction process (Maak and Pless, 2006). Current globalized and economic scenarios, organizational networks, and diversified workforces have put challenges on leaders. Leaders not only pay attention to increasing profits for shareholders but also endeavor to fulfill the needs of stakeholders (Miska et al., 2014; Han et al., 2019a). Various stakeholder demands, compelling tensions, and complex relation networks challenge the responsible leader to play a variety of roles in an organization (Maak and Pless, 2006). A responsible leader could be a housekeeper, dreamer, democratic negotiator, motivator, decision-maker, and discourser (Voegtlin et al., 2012; Wang et al., 2021). Responsible leaders always build and withstand profound relations among all the stakeholders by using the powerful forces of protection, acquisition, connection, and understanding (Lawrence and Pirson, 2015; Afsar et al., 2020). Responsible leadership places equal responsibility on all aspects of the organization and makes justified decisions through rational analysis of the interests of all the stakeholders for sustainable development, trust-building, and green action choices (Han et al., 2019a; Zhao and Zhou, 2019; Afsar et al., 2020).

Employees in organizations that practice paper-saving behavior, lower energy consumption, enhanced environmental protection, giving assistance to others in practicing green behavior, and recommending enhanced environmental protection are typical examples of OCBE (Afsar et al., 2020). Responsible leadership is a more complex and diverse leadership style than other traditional forms of leadership. The critical difference between other forms of leadership and responsible leadership is scope, values, society, environment, and positive change (Pless and Maak, 2011; Han et al., 2019b). Outmoded leadership styles exaggerate their influences but ignore the surrounding environment and overlook the interest of stakeholders. Other leadership styles also overlook the dimension of responsibility that is a crucial aspect of responsible leadership behavior (Voegtlin, 2016). On the other hand, the responsible leadership style focuses on complex stakeholder-leader relationships. A responsible leader takes care of the needs of various stakeholders besides protecting the interest of shareholders.

### Responsible Leadership and Organizational Citizenship Behavior for the Environment

Daily introduced the concept of Organizational Citizenship Behavior for the Environment (OCBE) in 2009; since then, it has been popular among management scholars. OCBE is a persons’ voluntary behavior toward the environment in the organization which is not covered by any formal incentive system (Daily et al., 2009). The voluntary behavior of OCBE includes reduced consumption of energy and resources, lowering carbon footprint, less usage of papers to save trees, helping colleagues, and proposing work suggestions in environment-friendly ways (Boiral and Paillé, 2012; Afsar et al., 2020). They further added that OCBE behavior works in three extents, i.e., eco-initiatives, eco-helping, and eco-civic engagements. Eco-initiatives are self-initiated and trivial steps that the individual takes to upkeep the environmental activities. Eco-helping is such a work setting in which colleagues help each other in activities that are pro-environmental. Moreover, eco-civic engagements are green activities in the workplace. These include steps and actions that contribute to the environment. OCBE fills the environmental gaps that are not identified and fixed by a formal system, by promoting complementarity and collaboration with ceremonial environmental management systems that cut the organizational costs on the environment and enrich the organizational reputation in terms of environmental concerns (Paillé et al., 2014; Alt and Spitzeck, 2016). Employees engaging in activities at an individual level, i.e. participation ability of employees, (Alt and Spitzeck, 2016), or engaging in organizational-level activities, i.e. pro-environmental atmosphere (Zientara and Zamojska, 2018; Afsar et al., 2020; Afshar Jahanshahi et al., 2021) promote OCBE.

Employees’ initiatives for improvements in organizational environmental performances are widely studied and incorporated in green literature (Boiral and Paillé, 2012). Employees’ actions directed toward environmental improvement are critically important (Daily et al., 2009). De Groot and Steg (2010) claimed that environmental-oriented actions addresses environmental issues and help the growth of an organization. The connection between OCBE and responsible leadership is better govern by the social learning theory. According to social learning theory, individuals shape their behaviors by observing and reproducing the behaviors of others (Bandura, 1986). Responsible leaders pay attention to the interests of different stakeholders of the business, including employees (Zhao and Zhou, 2019). When employees and followers observe the behavior of the leader, they gradually accept and reproduce this behavior. Responsible leaders take consideration of ethical issues and strive hard for up keeping relationships with stakeholders. OCBE is an individual’s ethical beliefs and actions that one takes to save the environment for oneself and society. This is why employees are inspired by responsible leaders, copy their environmental-friendly actions, and engage in OCBE.

Stahl and Sully de Luque (2014) claimed that a responsible leader encourages and reassures that organizations develop behavioral codes and measures that are related to the protection of the environment and clarifies environmentally friendly behaviors. Responsible leaders encourage employees for extra-role behaviors, as they take care of the interest of all the stakeholders within or outside the organizations, employees notice it, imitate the behavior of caring others, and perform extra-role activities that are primarily directed toward the environment (Han et al., 2019a; Zhao and Zhou, 2019). Responsible leaders conglomerate social responsibility with economic, social, and ecological benefits of all the stakeholders, including employees of the organizations that inspire them (Han et al., 2019a). Voegtlin et al. (2012) called this role model effect through which a responsible leader can motivate employees effectively to take the initiative for OCBE. Based on this relationship, we hypothesize:

***Hypothesis 1:*** *Responsible Leadership is positively associated with OCBE.*

### Responsible Leadership and Psychological Ownership

Psychological ownership is based upon psychological ownership theory and is defined by Pierce et al. (2001) as “the state in which individuals feel as though the target of ownership or a piece of that target is ‘theirs’ (i.e., ‘It is mine!’).” This is a state of mind in of employee that assumes the organization belongs to him. Pierce et al. (2001) claimed that psychological ownership depends upon three basic human needs, i.e., need of home or shelter, self-identity, and self-efficacy. The satisfaction of these basic instincts gives birth to a sense of psychological ownership. Some studies have conceptualized the four categories of psychological ownership as self-identity, self-efficacy, belongingness, and accountability (Pierce et al., 2001, 2003; Avey et al., 2009). The sense of psychological ownership toward organization make employees more proactive, caring, and attached to the organization with the sagacity of responsibility (Cheng et al., 2021). The responsible leadership style is based upon stakeholder relations and ethical consideration. This leadership style takes account of various stakeholder-leader relationships. This relationship is better to govern by the social exchange theory. According to Han et al. (2019b) emotional resources are exchanges in human societies. A responsible leader takes care of the financial, social, psychological, and environmental needs of various stakeholders, including employees, besides protecting the interest of shareholders (Zhao and Zhou, 2019). The leaders care and protection positively affect the employees and they feel obliged and an important part of the institution. So for this emotional exchange, employees, in return, develop feelings of ownership for the institution, and invest extra time and energy for the organization and collective welfare. It is also evident from the previous literature that responsible leadership behavior is considered a critical factor that has a positive association with psychological ownership of employees (Bernhard and O’Driscoll, 2011; Alt and Spitzeck, 2016; Kim and Beehr, 2017). A responsible leader takes care of the interests of employees and encourages them for their contribution to the process of decision making. It generates a sense of responsibility and accountability among followers. When employees are engaged in the decision-making process, they feel accomplished, show more attention, put extra efforts to complete tasks and obtain targets, and feel a sense of psychological ownership and responsibility for performance.

***Hypothesis 2:*** *Responsible Leadership is significantly associated with psychological ownership.*

### Psychological Ownership and Organizational Citizenship Behavior for the Environment

O’driscoll et al. (2006) also clarified that employees’ sense of ownership is positively associated with organizational citizenship behavior and motivates employees for extra-role behaviors. Understanding employees that the organization is “theirs” (psychological ownership) gives birth to the feelings of a part and owner of the organization, bearing in mind full responsibility of the organization, and striving hard for its sustainability. This pro-organizational affection and motivation make employees perform extra-role activities for the sustainability of the organization. OCBE is the voluntary actions of employees that are not governed by any formal reward system and directed toward environmental safety and sustainability. De Groot and Steg (2010) claimed that environmental-oriented actions address environmental issues and further help for the organization’s growth by saving water, reducing the usage of paper, reducing the consumption of electricity, and other green practices and procedures. The four dimension conceptualization of psychological ownership emphasized by Avey et al. (2009) includes self-identity, self-efficacy, belongingness, and accountability. This is why, if employees assume the organization as “theirs,” they consider the organization a significant part of theirs. The sense of belongingness and ownership, and they feel responsible and accountable for the organization’s sustainability. Psychological ownership makes employees think that if their organization is sustainable and prosperous, then employees are successful (Wang L. et al., 2019; Ullah et al., 2021). This ownership will breed extra motivation for their work, indulging voluntarily in extra-role activities that are beneficial for the employees, organization, and society, i.e., OCBE. Thus, employees with psychological ownership for the organization in mind will take care of the organizational sustainability and take initiatives directed toward organizational behavior directed toward the environment for the organization’s support, i.e., OCBE.

***Hypothesis 3:*** *Psychological ownership is significantly correlated with OCBE.*

### Mediating Role of Psychological Ownership

The responsible leadership style emphasizes upon stakeholder-leader approach. The responsible leader considers all the needs and demands of the stakeholders. All the social, psychological, financial, and environmental needs of various stakeholders are prioritized along with safeguarding shareholders and employees’ interests. This care and sense of protection ignite positivity and a sense of importance among employees. They consider themselves an essential part of an organization and, in return, establish a sense of psychological ownership for the organization. They consider themselves responsible for organizational success and sustainability and voluntarily invest extra time and energy to discharge the responsibility of psychological ownership (Avey et al., 2009; Bernhard and O’Driscoll, 2011; Kim and Beehr, 2017). The employees’ sense of accountability and psychological ownership motivate employees for the success and sustainability of organizations. Employees perform extra tasks other than their job description, complete extra-role activities, and indulge in such activities i.e., saving electricity, saving papers making green work environments (Wang L. et al., 2019; Afsar et al., 2020). Psychological ownership will raise motivation for job performance and extra-role activities that are good for the employees, organization, and society, i.e., OCBE. Thus, employees with psychological ownership for an organization in mind will take care of the sustainability of an organization will take more initiatives that will direct toward organizational citizenship behavior for an environment for the organization’s support. This discussion develops the hypothesis that:

***Hypothesis 4:*** *Psychological ownership mediates the relationship between responsible Leadership and OCBE.*

### Moderating Role of Employee Environmental Commitment Between Responsible Leadership and Psychological Ownership

Environmentally committed employees respond positively to signals from their organization regarding the environment and green practices (Cantor et al., 2012). The role of management is vital in the delivery of this signal. Managers who have strong knowledge of environmental issues and have control and decision-making powers are more practical to convince employees about environmental concerns (Robertson and Barling, 2013). Raineri and Paillé (2016) conveyed that sense of employee environmental commitment develops in employees who see their leader’s commitment with positivity and support their pro-environmental objectives. Psychological ownership is a sense or state of an employee in which they assume that organization belongs to them (Pierce et al., 2001). Employees consider organizational environmental objectives as their personal objectives. Thus, their personal environmental objectives and organizational environmental objectives align together (Afshar Jahanshahi et al., 2021).

The attitude theory of Bagozzi (1992) narrates that when employees’ are appreciated and valued either by the leaders or management and organization, they express positive tendencies and affirmative response in return. A responsible leader cares about the interest of stakeholders, including employees and organization, as well as gives priorities to environmental concerns (Zhao and Zhou, 2019). When environmentally committed employees are appreciated and valued by the responsible leaders, and their environmental objectives are aligned with organizational environmental objectives, their psychological ownership for the organization is enhanced. The environmental concern of responsible leaders inspires and aligns employees’ goals with the goals and objectives of their own, which are the objectives and mission of any organization. In this way, they increase their commitment and sense of psychological ownership for their organization. This discussion develops the next hypotheses that:

***Hypothesis 5:*** *Employee environmental commitment moderates the relationship between responsible leadership and psychological ownership such that the relationship will be stronger for those high in Employee environmental commitment.*

### Moderating Role of Employee Environmental Commitment Between Responsible Leadership and Organizational Citizenship Behavior for the Environment

Commitments have gained exceptional attention of management researchers as these are the gear of specific behavior to facilitate employees in the attainment of goals (Meyer and Herscovitch, 2001; Lawler et al., 2009), and a great deal of research related to workplace commitments has been done in the domain of organizational behavior (Cohen, 2003). In the literature of corporate greening, the role of commitment is widely studied as well (Keogh and Polonsky, 1998; Perez et al., 2009; Cantor et al., 2012). Commitment is an intuitively expressed mindset that provides behavioral directions toward any individual, idea, cause, or entity. Commitment toward a social or natural target, for example, the environment, is developed based upon normative and affective grounds (Meyer and Parfyonova, 2010; Bingham et al., 2013).

Employee environmental commitment is employee attachment and responsibility towards the environment at work (Raineri and Paillé, 2016). Daily and Huang (2001) claimed in their study that employee commitment towards an environment motivates employees to be involved in environmental friendly behavior at the workplace that is environment friendly. This is why employees who are environmentally committed take environmental initiatives and extra perform than their assigned duties. Morgan and Hunt (1994) also contributed that committed employees showed fewer intentions to leave and are more engaged with the organization, express more ownership, and exercise motivation. The environmentally committed employees perform extra-role behaviors, as they are more attached to the green mission of the organization and care about the environmental concerns of stakeholders (Srivastava and Dhar, 2016; Afsar et al., 2020). Similarly, other studies also found that commitment has an interconnection between particular behaviors that target environment, ownership, and organization (Erdogan et al., 2015; Montabon et al., 2016).

***Hypothesis 6:*** *Employee environmental commitment moderates the relationship between responsible leadership and OCBE such that the relationship will be stronger for those high in Employee environmental commitment.*

**FIGURE 1:**
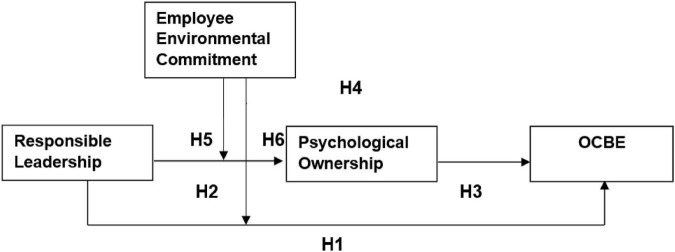
Model of this study.

The above mentioned relationships are portrayed in Figure 1, that shows the comprehensive model of the study and path of hypothesis development.

## Materials and Methods

### Research Context

The sample of the study includes 520 employees of six manufacturing and service organizations: an engine manufacturing plant, an insurance company, two petroleum manufacturing plants, and two banking offices located in Beijing, Sichuan, and Shaanxi provinces of China, considered as industrial provinces. Data was collected by convenient sampling technique. Recently, the Chinese government has paid great attention to green initiatives and developing a role for environmental protections. We considered these organizations for examining their organizational citizenship behavior for the environment and their work on green initiatives. These organizations are taking green initiatives and details are provided on their websites. For inclusion and exclusion purposes, we first ensured that organizations must be listed on stock exchanges, then organizations must have hundred or more employees, and the organizations had to be taking environmental problems seriously and providing details of green initiatives on their websites. As the target population was Chinese, for this purpose, the English version of the survey was translated into the Chinese language by two Chinese professors with fluency in English and Chinese languages. For the accuracy of the Chinese version of the survey, it was again translated back into English by similar Chinese professors (Brislin, 1980).

### Data Collection Procedure

The firms’ human resource departments were contacted by emails, using some references, and through telephonic contact. Data for the study was collected using an online survey on two points of time divided by the gap of 1 month to lessen the potential common method biases (Podsakoff et al., 2003). All the respondents were informed that the research was purely for academic purposes. The data were collected at two points of time. At the first point of time, data related to demographics of participants, responsible leadership, and psychological ownership were collected online through a survey. At the second point of time, we asked the employees to give their responses related to OCBE and employee environmental commitment.

### Measures

The measure of the study is divided into three parts. In the first part, the overview of the study is given and the importance of the role of responses is discussed. The purpose of the study is clarified to the respondents and it is further informed that the research was purely for academic purposes. In the second part, demographics are asked i.e., gender, age, education, and work experience of the respondents. In the third and main part of the questioner, items related to variables of the study were asked. The respondents answered the questionnaire on a five-point Likert scale from 1 (strongly agree) to 5 (strongly disagree). The Questionnaire was translated into the Chinese language to facilitate respondents.

#### Responsible Leadership

Responsible leadership measurement was assessed by using a five-item scale developed by Voegtlin (2011). The questions include (“My direct supervisor demonstrates awareness for the relevant stakeholder claims,” “My direct supervisor weighs different stakeholder claims before making a decision,” “My direct supervisor considers the consequences of decisions for the affected stakeholders,” “My direct supervisor involves the affected stakeholders in the decision-making process” and the last item is “My direct supervisor tries to achieve a consensus among the affected stakeholders”). The Cronbach’s alpha was 0.82 for the above-mentioned items.

#### Organizational Citizenship Behavior for the Environment

We used the 10 item scale of OCBE developed by Boiral and Paillé (2012). Sample items included but were not limited to (“In my work, I weigh the consequences of my actions before doing something that could affect the environment,” “I undertake environmental actions that contribute positively to the image of my organization,” and “I spontaneously give my time to help my colleagues take the environment into account in everything they do at work”). The Cronbach’s alpha of these items was 0.81.

#### Psychological Ownership

We assessed psychological ownership by the scale developed by Pierce et al. (1991). A five-item Likert scale was used, where 1 donated for “Strongly agree” and 5 donated for “Strongly disagree.” The sample items include (“I feel that I belong in this organization,” “For me, the organization is home,” “I am totally comfortable being in this organization,” “I feel that this organization’s success is my success,” and “I feel that being a member in this organization helps me realize my value”). The Cronbach’s alpha of these items was 0.83.

#### Employee Environmental Commitment

We used the eight-item scale of employee environmental commitment was developed by Raineri and Paillé (2016). The Cronbach’s alpha of this scale was 0.82. Sample items include (“The environmental concern of my company means a lot to me” and “I really care about the environmental concern of my company”).

## Analytical Strategy

In this study, data were examined using SPSS 25 and AMOS. The bootstrapping technique in SPSS 25 was also used in this study. Following Khan et al. (2019), the existing study used AMOS software for statistical analysis. Test the convergent and discriminant validity of the scales, the measurement model was established, convergent validity aims to investigate whether the items measure a similar concept or not. That contains composite reliability and average variance extracted. According to Hair et al. (2010) the average variance extracted (AVE) exceed the value of 0.50 and composite reliability (CR) exceed the value of 0.70 are accepted. To test the hypotheses’ structural model was established, coefficient values, confidence intervals, *P*-values, and t-statistics were calculated.

## Results

In six organizations approximately 750 employees were randomly selected, so a total of 750 survey links were distributed out of which 520 (69.33%) were received back completed in all senses and were used for data analysis. Out of these 520 respondents, 232 respondents (44.6%) were female, and 288 (55.4%) were male respondents. The age-wise distribution includes five age brackets. The first age bracket consists of age 20–25 and 71 respondents fall in this bracket. The second age bracket is 25–30 and a total 132 respondents fall in this age category. A total of 240 respondents, making up 46%, fall in the third age group of 30–35. Similarly, the fourth age group has an age limit 35–40, and a total 55 respondents fall in this age group, which is 10.6% of total respondents. The lowest number, i.e., 22 respondents, falls in the highest age group of above 40. The sample employees having work experience between 1 and 5 years are 30.2%, the largest group of employees, 45.4%, had work experience between 5 and 10 years, and the remaining 24.4% had work experience above 10 years. In employees’ education level distribution, 65.4% of respondents had a bachelor’s degree while 23.8% of respondents had a master’s degree. Supplementary information of the respondents is shown in below Table 1.

**TABLE 1:** Respondent’s demographic characteristics.

		Frequency	Percent	Valid percent	Cumulative percent
Gender	Male	288	55.4	55.4	55.4
	Female	232	44.6	44.6	100.0
Age	20–25	71	13.7	13.7	13.7
	25–30	132	25.4	25.4	39.0
	30–35	240	46.2	46.2	85.2
	35–40	55	10.6	10.6	95.8
	Above 40	22	4.2	4.2	100.0
Education	High school	8	1.5	1.5	1.5
	Senior high school	30	5.8	5.8	7.3
	Bachelor	340	65.4	65.4	72.7
	Master	124	23.8	23.8	96.5
	Others	18	3.5	3.5	100.0
Work experience	Up to 1 year	25	4.8	4.8	4.8
	1–5 years	157	30.2	30.2	35.0
	5–10 years	236	45.4	45.4	80.4
	10–20 years	85	16.3	16.3	96.7
	More than 20 years	17	3.3	3.3	100.0

First of all, screening of data was carried out to find out the missing values, any abnormal response of outliers, the test of differences, and the technique of common method variance. The Harman single factor test for common method bias was carried out to undertake the factor analysis (exploratory) by using SPSS 25. We consider all the variables (responsible leadership, OCBE, psychological ownership, and employee environmental commitment) for factor analysis. The highest covariance value was 36.40% (below 50%) indicated that the common method bias problem does not exist.

Table 2 present the value of Cronbach’s alpha is greater than 0.80, which indicates that the values of all factors are above 0.5 that are acceptable and composite reliability is higher than 0.80 (Fornell and Larcker, 1981), thus the adequate reliability for each item is ensured. The content validity of the scale of study is ensured through a comprehensive review of the literature and feedback received from researchers. All the items of the instrument were translated into the Chinese language to make them easily understandable for Chinese respondents and then all the items were translated back into the English language to ensure the validity of the contents. In confirmatory factor analysis, all the factors’ loadings are higher than 0.50 which indicates the convergent validity (Chau, 1997). According to Hair et al. (2010), the average variance extracted (AVE) exceeds the value of 0.50 and composite reliability (CR) exceeding the value of 0.70 are accepted. Multicollinearity was measured by variance inflation factors (VIFs) and tolerance. A VIF value of more than 4.0, or tolerance of <0.2, means there is an issue of multicollinearity (Hair et al., 2010).

**TABLE 2:** Convergent validity.

Variable	Items	Standardized factor loadings	Cronbach’s alpha	Composite reliability	Average variance extracted (AVE)	VIF
Responsible leadership	RL-1	0.850	0.905	0.928	0.722	2.736
	RL-2	0.851				2.898
	RL-3	0.823				2.536
	RL-4	0.863				2.879
	RL-5	0.860				3.091
Psychological ownership	PO-1	0.831	0.884	0.915	0.684	2.246
	PO-2	0.804				1.98
	PO-3	0.832				2.109
	PO-4	0.848				2.4
	PO-5	0.822				2.066
Employees environmental commitment	EEC-1	0.773	0.926	0.938	0.656	3.373
	EEC-2	0.816				4.118
	EEC-3	0.836				2.619
	EEC-4	0.821				2.554
	EEC-5	0.820				2.477
	EEC-6	0.794				2.045
	EEC-7	0.807				2.382
	EEC-8	0.811				2.394
Organizational citizenship behavior for the environment	OCBE-1	0.774	0.940	0.949	0.651	3.827
	OCBE-2	0.779				3.31
	OCBE-3	0.811				3.263
	OCBE-4	0.850				4.091
	OCBE-5	0.783				2.657
	OCBE-6	0.849				3.233
	OCBE-7	0.798				2.842
	OCBE-8	0.809				3.482
	OCBE-9	0.817				4.345
	OCBE-10	0.795				3.263

We used AMOS to assess the model fitness and hypothesized results. We built the model fitness around various statistical indices, such as χ^2^, CFI, TLI, and RMSEA following (Hair et al., 2010) and results are shown in Table 3. The CFI and TLI values within the range of 0.90–1.00 are considered good fit indices, whereas RMSEA values less than 0.05 and between 0.06 and 0.08 are deemed good fit and acceptable, respectively. The results depicted in Table 3 shows the following fit index values: χ^2^ = 187.843, χ^2^/*df* = 3.415, CFI = 0.972; TLI = 0.961; RMSEA = 0.068 which demonstrated that the fitness values are within the recommended ranges according to (Hair et al., 2010).

**TABLE 3:** Model fitness.

Model	X^2^	Df	X^2^/df	CFI	TLI	RMR	RMSEA
Model 3. Three factor Model	187.843	55	3.415	0.972	0.961	0.070	0.068
Model 2. Two factor model	595.583	129	4.617	0.935	0.923	0.069	0.083
Model 1. One factor	1723.945	330	5.224	0.896	0.881	0.084	0.090

***p < 0.001. N = 520.

*CFI, comparative fit index; RMSEA, root-mean-square error of approximation; TLI, Tucker-Lewis index; RL, Responsible leadership; PO, Psychological Ownership; EEC, Employees environmental commitment; OCBE, Organizational citizenship behavior for the environmental.*

Table 4 indicates means, standard deviation, and correlation among variables, and the results reveal that Pearson correlation among variables was positive and significant. Therefore other demographic variables such as age, gender, education, the organization indicated mean value with standard deviation and standard error, furthermore other constructs such as responsible leadership, psychological ownership, employees’ environmental commitment and organizational citizenship behavior for the environment indicated significant and positive correlations. The following table shows the mean values with standard deviation and standard error, the highest mean value is recorded for education followed by experience age and OCBE while lowest mean value is recorded for gender. Moreover gender is not significantly related with responsible leadership (RL), psychological ownership (PO), employees environmental commitment (EEC) and OCBE), while age has significant relationship with RL and OCBE but insignificant with EEC and PO. Education has significant relationship with all continuous variables such RL, PO, EEC and OCBE, further analysis of results revealed that experience significantly associated with RL and OCBE but insignificant with EEC and PO. In addition all continuous variables i.e. responsible leadership, psychological ownership, employee environmental commitment and OCBE are significantly related with each other at *p* < 0.01 level, and all values are significantly correlated (*r* = 0.331, *p* < 0.01) these results highlight responsible leadership and psychological ownership, and employees environmental commitment and responsible leadership are indicated (*r* = 0.328, *p* < 0.01). Employees’ environmental commitment and psychological ownership show a correlation (*r* = 0.885, *p* < 0.01). The OCBE shows positive correlations with all other constructs.

**TABLE 4:** Means, standard deviations, matrix for study variables.

Variables	Means	S.D.	1	2	3	4	5	6	7	8
1-Gender	1.45	0.498	1							
2-Age	2.66	0.982	−0.031	1						
3-Education	3.22	0.670	0.111*	0.153**	1					
4-Experience	2.83	0.873	−0.088*	0.815**	0.103*	1				
5-Responsible leadership	2.47	1.02	0.057	–0.243**	0.125**	–0.217**	1			
6-Psychological ownership	2.15	0.88	0.036	–0.060	–0.157**	–0.086	0.331**	1		
7-Employees environmental commitment	2.16	0.878	0.071	–0.034	–0.105*	–0.046	0.328**	0.885**	1	
8-OCBE	2.55	0.946	0.050	–0.225**	0.102*	–0.215**	0.891**	0.424**	0.452**	1

*N = 520.*

*p < 0.05, **p < 0.01, ***p < 0.001.

*SE, Standard error; SD, standard deviation.*

Table 5 represents the data of hypothesis testing that show that there is a significant and positive relationship between responsible leadership and OCBE (β = 0.298; *p* < 0.001), which provides support for our hypothesis H1. The table also shows that there is a significant and positive relationship exist between responsible leadership and psychological ownership (β = 0.284; *p* < 0.001) which also supports our hypothesis H2. Furthermore, it has been indicated that psychological ownership has a significant positive influence on OCBE and the results show that (β = 0.156; *p* < 0.001) psychological ownership changes organizational citizenship behavior to this extent. Therefore, these hypotheses H1 and H2, and also H3 are accepted in this study empirically, intensifying the significant relationship between responsible leadership, psychological ownership, and OCBE. There is also a significant mediating effect of psychological ownership on the association of responsible leadership and OCBE, as the table indicates (β = 0.238; *p* < 0.001), which provides support for mediating relationship and acceptance of H4. Furthermore, previously in the literature, we anticipated that employees environmental commitment would moderate the relationship between responsible leadership and psychological ownership. Besides supporting the moderation analysis, and the strength of indirect value (mediation), it is likely to rely on the value of moderation (i.e. psychological ownership) which is known as a conditional indirect effect or moderated mediation (Hayes and Rockwood, 2020). The overall results show partial mediation. Tables 6 and 7 exhibit the direct and indirect effect of responsible leadership on OCBE and Table 6 indicates a direct effect of 0.298 and Table 7 shows an indirect effect of 0.238.

**TABLE 5:** Hypothesis testing.

Hypotheses	Coefficient	S.D.	*T*-value	*P*-value	LLCI	ULCI
RL > OCBE	0.298***	0.056	5.26	0.000	0.187	0.409
RL > PO	0.284***	0.036	7.974	0.000	0.214	0.354
PO > OCBE	0.156***	0.022	0.019	0.000	0.114	0.199
RL > PO > OCBE	0.238***	0.017	4.705	0.000	0.152	0.325
RL* EEC > PO	0.063***	0.023	2.799	0.005	0.107	0.019
RL* EEC > OCBE	0.065***	0.021	2.949	0.003	0.022	0.108

ULCI, Upper level confidence interval; LLCI, Lower level confidence interval; SD, standard deviation; *p < 0.05, **p < 0.01, ***p < 0.001; RL; Responsible leadership; PO; Psychological Ownership; EEC; Employees environmental commitment; OCBE, Organizational citizenship behavior for the environmental.

**TABLE 6:** Direct effect.

Direct effect	Effect	S.D.	*T*-value	*P*-value	LLCI	ULCI
	0.298	0.056	5.26	0.000	0.187	0.409

*SD, Standard deviation; ULCI, Upper level confidence interval; LLCI, Lower level confidence interval.*

**TABLE 7:** Indirect effect.

Indirect effect	Effect	S.D.	*T*-value	*P*-value	LLCI	ULCI
	0.238	0.017	4.705	0.000	0.152	0.325

*SD, Standard deviation; ULCI, Upper level confidence interval; LLCI, Lower level confidence interval.*

Table 8 shows the values of standard error, and the bootstrap confidence interval of moderation conditional indirect effect of employee’s environmental commitment, respectively, low medium and the high level of employees environmentally commitment, the conditional indirect effect of employee’s environmental commitment significantly stronger and higher at the level of (0.1146) and significantly less strength at the lower level of (0.0177), we therefore found support for H5. Table 9 shows the conditional moderation effect of employees’ environmental commitment between responsible leadership and OCBE the higher level of value is (0.0032) and the lower is (0.005), so H6 is accepted.

**TABLE 8:** Results of the indirect conditional effect (moderation effect of EEC between RL and PO).

Moderator value	Effect	Bootstrap SE	Bootstrap LLCI	Bootstrap ULCI
1.5000	0.0017	0.0219	–0.0448	0.0414
1.7500	0.0177	0.0194	0.0204	0.0558
3.0000	0.1146	0.0291	0.0574	0.1718

*SE, Standard error; ULCI, Upper level confidence interval; LLCI, Lower level confidence interval; RL, Responsible leadership; PO, Psychological ownership. Number of bootstrap samples = 520; Level of Confidence = 95%.*

**TABLE 9:** Results of the conditional indirect effect (moderation effect of EEC between RL and OCBE).

Moderator value	Effect	Bootstrap SE	Bootstrap LLCI	Bootstrap ULCI
1.5000	0.0000	0.0010	0.0023	0.0022
1.7500	0.0005	0.0013	0.0039	0.0017
3.0000	0.0032	0.0064	0.0178	0.0084

*SE, Standard error; ULCI, Upper level confidence interval; LLCI, Lower level confidence interval; RL, Responsible leadership; OCBE, Organizational citizenship behavior for the environment.*

*Number of bootstrap samples = 520; Level of Confidence = 95%.*

**FIGURE 2:**
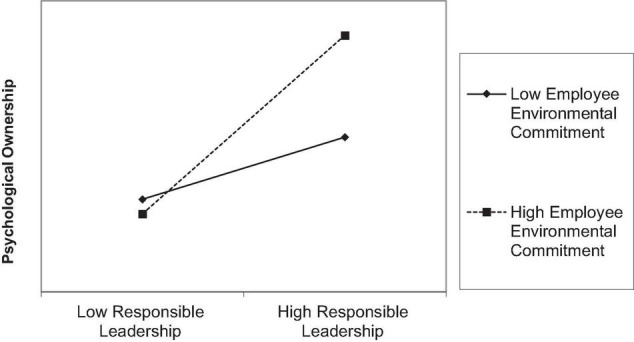
Interaction chart.

Figure 2 depicts an interaction chart that shows the moderation effect. It shows two lines interacting with each other at a point, so it indicates that there is moderation between responsible leadership and psychological ownership.

## Discussion

According to Voegtlin et al. (2012), responsible leadership is the most emerging and compensated leadership to enhance the environmental reputation and maintain sustainable development of the organization and society. The stakeholder theory elaborates responsible leadership, corporate social responsibility (CSR), and leadership ethics. Past studies of Han et al. (2019a) and Ullah et al. (2021) indicated that responsible leadership positively impacts OCBE. In this study, by performing path analysis we attempted to answer the relationship of responsible leadership with OCBE. How does responsible leadership support OCBE on the basis of social learning theory? Based on mediation and moderation, we developed a conceptual model that included psychological ownership and employees’ environmental commitment. The results indicated that responsible leadership behavior provides support for OCBE. In our research environment, specific responsible leadership was found to foster employees through psychological ownership mediation. Additionally, employees’ environmental commitment plays a moderating role to intensify the effect.

This study adds psychological ownership to differentiate these two mechanisms with employees’ environmental commitment as a control moderator. The result showed that psychological ownership mediates the relationship between responsible leadership and OCBE. In practice, the results indicated some new suggestions on how to encourage employees to actively engage in OCBE within the organization. Furthermore, this study indicated a partial mediation in the table of mediation, and the results showed a significant linkage of psychological ownership playing a mediator role between responsible leadership and OCBE. These findings are in line with previous studies (Han et al., 2019a; Zhao and Zhou, 2019; Ullah et al., 2021), which argued that responsible leadership is essential for promoting sustainability-related behavior among employees. This is why because responsible leaders can lead existing employees to strive for sustainable priorities and values, particularly development. In return, employees engage in sustainable practices, i.e., pro-environmental behaviors. As compared to the traditional leadership behaviors and leader-follower perspectives, responsible leadership is more helpful in improving sustainable personal behaviors. Furthermore, employee environmental commitment positively moderated the mechanism linking responsible leadership and psychological ownership such that this indirect influence was significantly positive. Our study observed a moderating effect that influences the relationship between responsible leadership and psychological ownership. Furthermore, employees’ environmental commitment plays a moderating role that intensifies the relationship between responsible leadership and OCBE.

## Conclusion

According to Voegtlin et al. (2012), responsible leadership is one of the most emerging and compensated leadership styles to enhance the repute of an organization and maintain the sustainable development of the organization and of society. OCBE describes the employee’s behavior related to environmental protection that is not governed by any organization’s formal reward system. Principally, it is employees’ optional behavior directed toward environmental protection and epitomizes an operative supplement for the peoples’ environmental safeguarding behavior and the green growth approaches of the organizations (Daily et al., 2009).

In this research, we endeavor to investigate the association between responsible leadership and OCBE in the mechanism of psychological ownership and employee environmental commitment. The sample used for data analysis of this study consist of 520 employees from Chinese organizations. For data analysis the SPSS 25 and AMOS used and drew the following conclusions; responsible leadership positively and significantly affects the OCBE. Psychological ownership performed a mediating role in the relationship of responsible leadership and OCBE, employee environmental commitment plays a moderating role between responsible leadership and psychological ownership, and OCBE.

This study contributed theoretically to social learning and social exchange theories. The social learning theory strives for the compound behavior of people which is primarily acquired through direct and indirect observation and imitation of the behavior of activists or objects (Bandura, 1986). In the study of Maak and Pless (2006), responsible leadership is described as internal and external stakeholder protection with a diverse range of associates within the enterprise and outside the enterprise i.e natural environment. We investigate and enrich the literature by examining the association between employee environmental protection behavior and responsible leadership, i.e., OCBE. We proved that responsible leadership develops a sense of responsibility and inspires employees to encompass environmental protection behavior. This research also emphasizes the effect of responsible leadership on the employees’ behavior and attitudes through employees’ sense of psychological ownership for the institutions and employee environmental commitment for environmental protection.

### Theoretical Implications

This study contributed to the existing literature of responsible leadership, organizational citizenship behavior, psychological ownership, and employee environmental commitment. Data for the study were collected from three provinces of China. This study contributed to the existing body of knowledge of responsible leadership and organizational citizenship behavior based upon social learning theory. The study proves that to nurture organizational citizenship behavior for the environment, the importance of responsible leadership is undeniable. In the emerging environmental issues, particularly in developing countries like China, leaders having the traits of responsible leaders can motivate employees for socially responsible behavior that is beneficial not only for themselves, but also for the organization and for the environment at a large. Furthermore, this study also sheds new light on the existing model of responsible leadership and OCBE in the connection of employees’ psychological ownership. Additionally, based upon social exchange theory, this study analyzes mediating effects of psychological ownership on the primary relationship of OCBE and responsible leadership. This study underwrote the efforts in the Chinese context and expanded the context of social exchange theory. Han et al. (2019b) argued that emotional resources are exchanges in human societies. The study at hand supported their argument by adding that the qualities of a responsible leader encourage followers, for a particular behavior for example OCBE, and the presence and exchange of essence of ownership increased this behavior. Lastly, this work extended the literature of employee environmental commitment in the eastern context. Some previous studies (Jiang et al., 2019; Mi et al., 2019; Tuan, 2019; Pham et al., 2020) investigated the multiple leadership styles for organizational citizenship behavior of employees for the environment and tested multiple moderation effects, but the environmental commitment of employees was not included. This study endeavors and try to fill the gap by including employee environmental commitments as a moderator for responsible leadership and psychological ownership.

### Management and Policy Implications

This paper investigated the role of responsible leadership for organizational citizenship behavior for the environment in the Chinese context with the mediation and moderation of psychological ownership and employee environmental commitment. Based upon the findings of the current study, we draw the following practical implications for management and policymakers. First, the role of responsible leadership for nurturing the organizational citizenship behavior for the environment in the organization should be highlighted. In an organizational setting, the managers’ sense of CSR and environmental-related ethics affect the employees’ practices and attitudes. So, the level of traits of responsible leadership in managers should be improved. Managers should be hired who possess the characters of responsible leadership, or managers should undergo training programs that produce and polish such characteristics. The manager’s collaboration with employees should be encouraged, to stimulate employee workplace environmental protection attitudes and practices and their efforts for organizational sustainable development. Second, organizations should hire such leaders and develop leadership training programs that demonstrate the ethics and characteristics of responsible leadership. These ethics and values should be embedded in training and leadership development programs to support responsible leadership behavior. Furthermore, organizations should encourage the employee’s pro-environmental behavior by endorsing environmental-friendly attitudes and practices like reducing carbon emissions, saving energy, and reusing resources.

Third, employee psychological ownership affects employees’ motivation to involve in environmental protection behavior. To increase the level of OCBE in the organization, it is essential to stimulate their sense of psychological ownership for the organization. In return, the employee will exhibit environmental protection behavior. Psychological ownership gives employees a sense of ownership in the organization, so they feel more attached to the organization, strive for its sustainable development, and in this way show environmental protection behavior. Therefore, training programs should be implemented to enhance the employees’ sense of psychological ownership for the organization and increase their skills to participate in environmental protection activities.

### Research Limitations and Future Directions

There are a few limitations associated with this study to be deliberated. First of all, the instrument used to measure responsible leadership is derivative from scales developed for the western perspective. Scales have good validity and reliability, but the theoretical association of responsible leadership and its endorsement for diverse cultures, particularly the eastern perspective, including China, needs further exploration. Second, although the use of the time-lagged data reduces the chances of common method bias, it prevents any causative inferences. It is recommended for upcoming research to use longitudinal study designs to generate casual relationships. Third, in this study, psychological ownership was used as a mediator and employee environmental commitment was used as a moderation agent between responsible leadership and OCBE. The role of HR in developing green culture and climate as suggested by Luu (2019) should be further investigated in upcoming research. Fourth, in upcoming studies, the Chinese perspective should be further enlarged and include other eastern countries to examine and enlarge the eastern attitude on environmental issues. Fifth, data for leadership perspective was evaluated by the employees and not by the leader themselves. In future studies, we call for leaders’ self-evaluation of leadership traits and their impact on employees OCBE.

## Data Availability Statement

The datasets generated for this study are available on request to the corresponding author.

## Author Contributions

AA contributed to the data curation, formal analysis, conceptualization, and original draft and revision of the manuscript. YC contributed to the supervision and guidelines. Bilal contributed to the revision of the manuscript. SZ contributed to the review. SM contributed to formatting, review and editing of the manuscript. IU contributed to the, formal analysis, and revision of the manuscript. YHM contributed to the reviewed and edited of the manuscript. All authors contributed to the article and approved the submitted version.

## Conflict of Interest

The authors declare that the research was conducted in the absence of any commercial or financial relationships that could be construed as a potential conflict of interest.

## Publisher’s Note

All claims expressed in this article are solely those of the authors and do not necessarily represent those of their affiliated organizations, or those of the publisher, the editors and the reviewers. Any product that may be evaluated in this article, or claim that may be made by its manufacturer, is not guaranteed or endorsed by the publisher.
